# Editorial: Reverse Vaccinology

**DOI:** 10.3389/fimmu.2019.02776

**Published:** 2019-12-03

**Authors:** Richard Moxon, Pedro A. Reche, Rino Rappuoli

**Affiliations:** ^1^Department of Paediatrics, University of Oxford, Oxford, United Kingdom; ^2^Department of Immunology & O2, Complutense University of Madrid, Madrid, Spain; ^3^GSK, Siena, Italy; ^4^Faculty of Medicine, Imperial College, London, United Kingdom

**Keywords:** infectious diseases, vaccines, reverse vaccinology, microbiology, vaccinology

For many, the semantics of the term “*reverse vaccinology*” may be puzzling. Literally, it implies a complete change of direction or action in the study of vaccines. The non-obvious point is that this *volte face* first came about through whole genome sequencing (WGS). WGS revolutionized biology, including microbiology. Specifically, it introduced a top-down, computer data-based approach to the discovery of candidate vaccine antigens; highly sensitive, but not specific and, crucially, not hypothesis driven. This contrasted with the classical laboratory based, hypothesis driven analysis of microbes to identify components that could elicit protective immunity. *Reverse vaccinology* relies on the use of computational methods and tools to identify vaccine candidates for further experimentation, refinement of which is crucial for their optimal use as argued and detailed by Dalsass et al. These computational tools serve to anticipate antigens that are likely to induce protective responses as well as the precise antigen regions, epitopes, recognized by the immune system (1).

*Reverse vaccinology* was first used to predict potential antigens for a vaccine against the B strains of *Neisseria meningitidis* (meningococci) in the 1990's, reviewed by Masignani et al. It is worth emphasizing that the formulation of this complex vaccine could not have been achieved without the systematic, WGS-based approach to population biology succinctly captured in the review by Maiden, a pioneer in laying the foundations of the molecular epidemiological tools so crucial to the design of vaccines both for infectious and non-infectious diseases. More recently, Bianconi et al. have been successful in applying the classical *reverse vaccinology* approach for *Pseudomonas aeruginosa*. Starting from the 5,570 open reading frames of the genome, they selected 52 vaccine candidates by applying a number of filters to exclude the proteins predicted not to be present on the bacterial surface, to be variable in different strains, to have homology to human proteins, or to be homologous to *E. coli* proteins. Of the 52 predicted vaccine antigens, 30 were successfully expressed and several of those gave a quite remarkable protection in the mouse challenge model. However, one of the main aims of this current series on *reverse vaccinology* is to highlight how many new concepts and technologies have been recruited to facilitate vaccine design including contributions from proteomics, immunology, structural biology, systems biology, and mathematical modeling. Thus today, the change of direction and action in vaccine research, captured in the term *reverse vaccinology*, embodies much more than innovation in antigen discovery.

Bidmos et al. describe the isolation and recombinant expression of the variable regions of heavy (VH) and light (VL = κ or λ) chain genes of immunoglobulin (IgG) using a variety of molecular tools. Referred to as *reverse vaccinology 2.0*, this permits the high-throughput screening of large numbers of antibody-secreting cells and was employed to identify functional anti-*Staphylococcus aureus* monoclonal antibodies induced during bacteremia (2) and anti-MTB surface antigen antibodies cloned from patient-derived plasmablasts of reactivated memory B-cell origins (3). In a further study, Bidmos et al. describe successful efforts to utilize the *reverse vaccinology 2.0* approach to identify novel functional meningococcal antigens with the potential to expand the coverage of currently licensed meningococcal B vaccines.

The synergism of immune-information and systems immune-biology with WGS provides crucial tools that consider not just the challenges of the identification and molecular diversity of target antigens, but the importance of expression levels and how these variables, along with host genetic variation, impact on B-cell immune responsiveness. Immunologists do their best to identify the optimal epitopes of antigens for candidate vaccines, as exemplified by the work of Nagpal et al. These authors applied an immunoinformatic pipeline that led to identifying epitope-based vaccine candidates against 14 pathogenic bacteria and made them available through a web-resource named VacTarBac. Bacteria are complex pathogens encompassing numerous protein antigens that when targeted for epitope prediction will result in a huge number of candidates. But, this plethora of information and the challenge of what can be reasonably subjected to further rigorous investigation is a daunting challenge. Thereby, to simplify further experimental advances, the authors implemented a system to identify and prioritize virulence factors or other essential genes required for pathogenicity while also discarding epitopes cross-reactive with self-proteins. The application of stringent prioritization criteria to the selected 14 pathogenic bacteria led to the identification of just 252 unique B-cell and T-cell epitopes.

T-cell epitopes can be predicted starting from WGS. Tian et al. show how they made a full map of the T-cell epitopes starting from the 4,000 open reading frames of *Mycobacterium tuberculosis*. A metric (immunogenicity score) was devised based on predictions of their immunodominance, promiscuity, HLA restriction and conservation. In a second example, they describe how the prediction of the T-cell epitopes of *Bordetella pertussis* antigens, not just those included in currently licensed acellular vaccines, may help to design novel formulations based on Th1 and Th17 immunity to overcome the limitations of the existing vaccines which induce mostly a Th2 based immunity. Degoot et al. describe a new method to predict peptide binding to major histocompatibility complex class two (MHC-II) molecules, which is the main basis to anticipate CD4 T cell epitopes. The method is based on structural analyses of peptide-MHC II interactions and can predict peptide binding for all three human MHC-II loci (HLA-DR, HLA-DP, and HLA-DQ). The authors report that the performance of the method is in general comparable to neural network methods and is superior in predicting peptide binding to HLA-DP molecules. The main advantage of this approach reported over other machine learning models is that of being rooted on actual physicochemical peptide-MHC-II binding interactions. A main handicap is however that the authors have not made available the method for rigorous independent comparisons.

Sánchez-Ramón et al. makes a well-argued case for trained-immunity based vaccines (TIbV). These are vaccines that induce an innate, non-specific immunity for long periods of time. A typical example of a TIbV Vaccine is BCG which induces two types of immunity, one based on adaptive immunity specific for *Mycobacterium tuberculosis*, and the other based on innate immunity which is non-specific but so effective that it is also recommended to cure prostate cancer. This immunity induces activation of dendritic cells, activation of non-specific effector responses of innate immune cells such as monocytes and macrophages and is maintained overtime by epigenetic changes. Vaccine adjuvants and non-toxic derivatives of toxins (4) inducing non-specific protection against bacteria or viruses can be considered a proxy of TIbV.

Buckley et al. describe modeling approaches that provide exciting insights into AS01, one of the most successful adjuvants licensed for human use, and how such an adjuvant may work. According to Sánchez-Ramón et al., in addition to adaptive immune responses, it is also likely to induce trained immunity. AS01 has been licensed for the RTS-S vaccine against malaria and the Shingrix vaccine against Shingles and is part of the first successful clinical trial showing protection from disease in people infected by *Mycobacterium tuberculosis*.

A quarter of a century after WGS revolutionized biology, this series is a timely and exciting opportunity to reflect on what has been achieved by *reverse vaccinology* and how best to galvanize future efforts to improve global public health through rigorous and imaginative exploitation of the explosion in technologies that can be used to develop a broad range of novel vaccines.

## Author Contributions

All authors listed have made a substantial, direct and intellectual contribution to the work, and approved it for publication.

### Conflict of Interest

RR was employed by GSK and RM holds a consultancy agreement as a scientific adviser to GSK. The remaining author declares that the research was conducted in the absence of any commercial or financial relationships that could be construed as a potential conflict of interest.
